# NAction! How Can Neuraminidase-Based Immunity Contribute to Better Influenza Virus Vaccines?

**DOI:** 10.1128/mBio.02332-17

**Published:** 2018-04-03

**Authors:** Florian Krammer, Ron A. M. Fouchier, Maryna C. Eichelberger, Richard J. Webby, Kathryn Shaw-Saliba, Hongquan Wan, Patrick C. Wilson, Richard W. Compans, Ioanna Skountzou, Arnold S. Monto

**Affiliations:** a Center for Research on Influenza Pathogenesis (CRIP), New York, New York, USA; b Icahn School of Medicine at Mount Sinai, New York, New York, USA; c Centers of Excellence for Influenza Research and Surveillance (CEIRS)‡; d Department of Viroscience, Erasmus Medical Center, Rotterdam, the Netherlands; e Center for Biologics Evaluation and Research, Food and Drug Administration, Silver Spring, Maryland, USA; f St. Jude Center of Excellence for Influenza Research and Surveillance, Memphis, Tennessee, USA; g Department of Infectious Diseases, St. Jude Children’s Research Hospital, Memphis, Tennessee, USA; h Johns Hopkins Center of Excellence for Influenza Research and Surveillance, Baltimore, Maryland, USA; i Department of Emergency Medicine, Johns Hopkins University School of Medicine, Baltimore, Maryland, USA; j W. Harry Feinstone Department of Molecular Microbiology and Immunology, Johns Hopkins Bloomberg School of Public Health, Baltimore, Maryland, USA; k New York Influenza Center of Excellence (NYICE), New York, New York, USA; l Department of Medicine, the Knapp Center for Lupus and Immunology Research, Section of Rheumatology, the University of Chicago, Chicago, Illinois, USA; m Emory-UGA Center of Excellence for Influenza Research and Surveillance, Atlanta, Georgia, USA; n Department of Microbiology and Immunology, Emory University School of Medicine, Atlanta, Georgia, USA; o Emory Vaccine Center, Emory University School of Medicine, Atlanta, Georgia, USA; p Department of Epidemiology, University of Michigan School of Public Health, Ann Arbor, Michigan, USA; University of Texas Health Science Center at Houston

**Keywords:** influenza vaccines, neuraminidase, universal influenza virus vaccine

## Abstract

Neuraminidase is one of the two surface glycoproteins of influenza A and B viruses. It has enzymatic activity that cleaves terminal sialic acid from glycans, and that activity is essential at several points in the virus life cycle. While neuraminidase is a major target for influenza antivirals, it is largely ignored in vaccine development. Current inactivated influenza virus vaccines might contain neuraminidase, but the antigen quantity and quality are varied and not standardized. While there are data that show a protective role of anti-neuraminidase immunity, many questions remain unanswered. These questions, among others, concern the targeted epitopes or antigenic sites, the potential for antigenic drift, and, connected to that, the breadth of protection, differences in induction of immune responses by vaccination versus infection, mechanisms of protection, the role of mucosal antineuraminidase antibodies, stability, and the immunogenicity of neuraminidase in vaccine formulations. Reagents for analysis of neuraminidase-based immunity are scarce, and assays are not widely used for clinical studies evaluating vaccines. However, efforts to better understand neuraminidase-based immunity have been made recently. A neuraminidase focus group, NAction!, was formed at a Centers of Excellence for Influenza Research and Surveillance meeting at the National Institutes of Health in Bethesda, MD, to promote research that helps to understand neuraminidase-based immunity and how it can contribute to the design of better and broadly protective influenza virus vaccines. Here, we review open questions and knowledge gaps that have been identified by this group and discuss how the gaps can be addressed, with the ultimate goal of designing better influenza virus vaccines.

## INTRODUCTION

**FIG 1:**
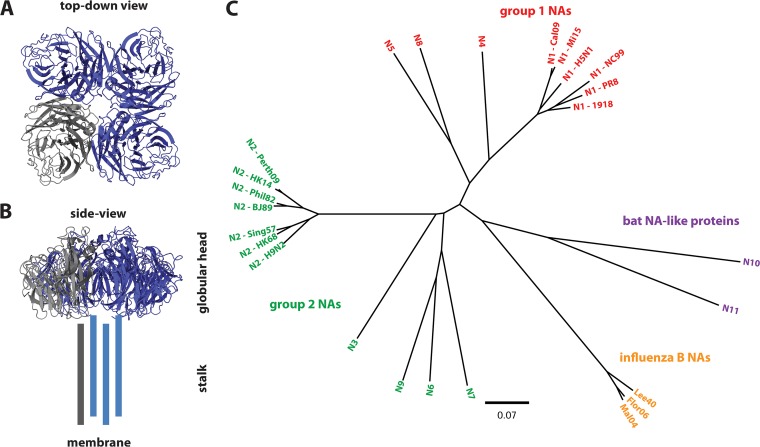
Influenza virus neuraminidase structure and phylogeny. (A) Top-down view of the NA tetramer, with one of the monomers indicated in gray. (B) Side view of the molecule. The structure of the variable stalk domain has so far not been solved and is indicated by the 4 bars. Panels A and B are based on the structure of the N2 NA of A/Tanzania/205/2010 (PDB number 4GZO [102]), visualized in PyMOL (Schrödinger, Inc.). (C) Tree of known influenza virus NAs and NA-like proteins (N10 and N11). Influenza NAs cluster into group 1 (N1, N4, N5, N8) and group 2 NAs (N2, N3, N6, N7, N9). Influenza B NAs as well as the NA-like proteins (from sequences found in bats) form their own clusters. The tree was generated using Clustal Omega and was visualized in FigTree. The scale bar represents a 7% amino acid difference. NA sequences from the following strains were used: A/California/04/2009 (N1-Cal09), A/New Caledonia/20/1999 (N1-NC99), A/Puerto Rico/8/1934 (N1-PR8), A/Vietnam/1203/2004 (N1-H5N1), A/Michigan/45/2015 (N1-Mi15), A/Brevig Mission/1/1918 (N1-1918), A/Hong Kong/1/1968 (N2-HK68), A/Hong Kong/4801/2014 (N2-HK14), A/Singapore/1/1957 (N2-Sing57), A/chicken/Hong Kong/G9/1997 (N2-H9N2), A/Philippines/2/1982 (N2-Phil82), A/Beijing/353/1989 (N2-BJ89), A/Perth/16/2009 (N2-Perth09), A/swine/Missouri/4296424/2006 (N3), A/mallard/Sweden/24/2002 (N4), A/mallard/Sweden/86/2003 (N5), A/Caspian seal/Russia/T1/2012 (N6), A/harbor seal/Germany/1/2014 (N7), A/Jiangxi-Donghu/346/2013 (N8), A/Hong Kong/125/2017 (N9), A/yellow-shouldered bat/Guatemala/060/2010 (N10), A/bat/Peru/33/2010 (N11), B/Lee/1940 (Lee40), B/Florida/04/2006 (Flor06), B/Malaysia/2506/2004 (Mal04).

Influenza A and B viruses express two surface glycoproteins which have essential functions in the viral life cycle. The more abundant glycoprotein is the hemagglutinin (HA), a type I transmembrane protein. HA exists as a trimer and mediates binding of the virus to host cells via interactions between its receptor binding site and the terminal sialic acids on host cell glycans. Once the virus is taken up into the endosome, HA also triggers fusion of viral and endosomal membranes after endosome acidification (1, 2). Many antibodies that target the HA are neutralizing because they block the ability of the receptor binding site of HA to interact with sialic acids on the host cell surface, thus preventing attachment and entry. Because protection was classically recognized to be related to anti-HA antibodies, currently licensed vaccines are designed to induce antibodies against HA (3). The development of better, more broadly protective vaccines is also mostly focused on the HA (4). However, the second surface glycoprotein, neuraminidase (NA), might also play a key role in the development of a better influenza virus vaccine. NA is a tetrameric type II transmembrane protein with an enzymatic function that cleaves terminal sialic acid from glycans on the host cell surface, a process termed receptor-destroying activity (5, 6) (see Fig. 1 for an overview). This activity is very important at several stages of the viral life cycle. As influenza viruses enter a host, they need to penetrate mucosal barriers in order for HA to reach the sialic acids on the host cell surface (7–9). Further, mucosal fluids contain natural defense proteins, such as mucins, that are heavily glycosylated, acting as a decoy for HA binding, which neutralizes the influenza virus (10). NA, however, has been shown to release virus particles and allow them to efficiently reach their host cells (7, 8, 11). Once a virus has successfully entered and replicated in the host cell, virus particles containing both HA and NA bud from the cell membrane. As the host cell surface contains sialic acid, HA of nascent virus particles adheres to the same host cell, preventing the release of the new particle. However, NA counteracts this by removing terminal sialic acids from the host cell, thus allowing the release of the nascent viral particles (12). Finally, viral particles adhere to each other by interactions between HA and sialic acid on glycans on HA or via other glycoproteins in mucus that act as adapters. NA counteracts this aggregation and might consequently enhance the spread and perhaps transmissibility of the newly produced virus through this activity by increasing the opportunity for virus particles to be transmitted in very small aerosol droplets (6). In addition, NA might play a role in viral entry as well (13, 14). NA activity is therefore very important for virus fitness and has been the target of licensed small-molecule drugs, like oseltamivir (15). The fact that inhibition of NA activity has a prophylactic and therapeutic effect validates NA as a target. However, NA has largely been ignored for vaccine development. A better understanding of NA-based immunity and its mechanisms of action might greatly contribute to the design of better, longer-lasting, and more broadly protective vaccines. In this position paper, the NAction! group, an NA focus group that was recently formed at a meeting of the Centers of Excellence for Influenza Research and Surveillance (CEIRS; funded by the National Institute of Allergy and Infectious Diseases) in Bethesda, MD, tries to identify the gaps in knowledge about NA immunity and its protective effects and lays out a path forward to address them.

## WHAT DO WE KNOW ABOUT NEURAMINIDASE-BASED IMMUNITY?

As outlined above, NA plays an important role in the virus life cycle, and targeting this protein by antibodies inhibits virus replication. NA antibodies are usually not capable of inhibiting virus entry into host cells but act at later stages of the life cycle when the virus buds from infected cells. As a result, plaque size and number are not reduced when NA-specific antibodies are included during infection in an *in vitro* plaque reduction neutralization assay. However, plaque size (diameter) is impacted when these antibodies are included in the agar overlay of the assay (16–19). Antibodies that inhibit NA activity at low concentrations are so effective in preventing virus spread that plaques are often not visible. Technically speaking, this means that most anti-NA antibodies are not capable of completely neutralizing the virus *in vitro*. However, it is well known that many anti-NA antibodies can inhibit the enzymatic activity of NA in *in vitro* NA inhibition (NI) assays (20), and this activity usually correlates well with a reduction of plaque size, as discussed above (17). We also know that the induction of a strong antibody response against NA in animal models can prevent clinically overt disease, while often not leading to sterilizing immunity (21–26). From human challenge studies performed in the 1970s, we know that anti-NA antibody titers correlate inversely with virus shedding and disease symptoms (27, 28). This means that virus replication is controlled and that no clinical disease is observed, but low-level virus replication is typically present. More recent clinical trials showed that NI titers are a correlate of protection against influenza virus-induced disease that is independent of HA-based immunity (29). This was further confirmed in direct human challenge studies (30). It is possible that N2-based immunity (induced by previous H2N2 infections) might have played a role in protecting subjects from pandemic H3N2 infections in 1968 (28, 31, 32). N2-based cross-reactivity has even been implied to be a factor in the extinction of H2N2 in 1968 after the emergence of H3N2 (33). We also know that NA undergoes evolution and can antigenically drift (34), but we have very limited knowledge about antigenic sites/epitopes that might be targeted by the immune system (16, 18, 35–41). Vaccines which contain HA and NA in close association may not induce anti-NA (42) responses that are as strong as those induced by NA given on its own (23, 24, 43, 44). Finally, we know that current inactivated influenza virus vaccines contain NA of variable quality and (nonstandardized) quantity, potentially with lot-to-lot variability, and these vaccines do not reliably induce robust anti-NA immunity (23, 34, 45–48). Nevertheless, some increases in NI antibody responses, although at low seroconversion rates and with a low fold induction, have been reported for most inactivated virus vaccines and for live attenuated influenza virus vaccines (LAIV), and the response rate and antibody titers are increased by vaccinating with high-dose formulations (48–50). This information, together with the finding that HA inhibition (HI) and NI titers correlate with protection against influenza virus infection independently (29, 51), shows not only that NA-based immunity is important but also that it is feasible to develop vaccines that produce consistently protective NA-specific antibodies. To conclude, we have evidence that NA-based immunity can be important and protective. However, there are many knowledge gaps that need to be addressed in order to move forward toward a rationally designed vaccine that induces robust and protective anti-NA immunity.

## WHAT DO WE NEED TO KNOW?

In order to close the current knowledge gaps, assess the role that NA-based immunity might play in protection from influenza virus infection, and design vaccines that induce robust anti-NA responses, the following actions should be taken.

### NA-based protection.

As mentioned above, studies by Couch et al. (27, 51), Murphy et al. (28), Monto et al. (29), and Memoli et al. (30) have shown that anti-NA antibody titers correlate with protection from disease in humans. These studies need to be expanded, the results need to be confirmed across H1N1, H3N2, and influenza B viruses for different age groups (which have different preexposure histories, etc.) and study designs, and the titers that correlate with protection need to be defined. Considering that NA antibodies control the spread of virus to and from epithelial cells, it will be important to understand the contribution of NA-specific immunity at mucosal surfaces. Also, the antigenic drift of NA should be studied in this context.

### Imprinting.

The phenomenon of immunologic imprinting and how it shapes immune responses to influenza virus HAs later in life is currently a major research focus. Imprinting should also be studied for NA, ideally by following subjects longitudinally from birth to determine how their immunity to NA is shaped by initial and sequential exposures to influenza virus and vaccines.

### Target epitopes.

Our understanding of which epitopes are targeted by human antibodies is limited at best. Human monoclonal antibodies induced by both vaccination and infection should be isolated and characterized. Antigenic sites need to be defined, and antibody footprints should be confirmed by structural biology methods.

### NA quality and quantity in vaccines.

Inactivated vaccines contain NAs of varied quality and quantity (23, 52). Very little is known about the stability of NA during the production process and over the shelf life of vaccines. NA activity is representative of the native structure and is an excellent measure of the ability of NA to induce NI antibodies as long as NA inhibitors (e.g., EDTA) are not present in the vaccine formulation (53). While a preliminary study suggests that NA activity might be maintained over vaccine shelf-lives for seasonal vaccines, the levels of stability of NAs from different strains vary (53). The NA quantity in currently licensed vaccines is insufficiently quantified and not standardized in the United States. However, new vaccine platforms are considering the NA content of pandemic vaccines, and NA immunogenicity is being measured in clinical trials more often (54, 55). Our understanding of NA immunogenicity would be greatly advanced if the NA contents of seasonal and pandemic vaccines were a required measure for batches used in clinical trials, and this should certainly be done in the future. It is important to make assays and reagents for these quantifications widely available (within and beyond the CEIRS network).

### Better vaccine formulations and strategies.

We already know that current inactivated vaccines induce varied anti-NA responses (approximately 30% seroconversion) (23, 34, 45–48). New vaccine formulations and strategies that induce a more robust anti-NA immune response should be developed and tested in clinical trials. This could include spiking the regular inactivated vaccines with purified NA or giving purified NA in addition to current vaccines (3, 56). A clinical trial with NA purified from H3N2 virions demonstrated that NA is safe and immunogenic, with a dose of 7.7 µg inducing a 3-fold or greater increase in NI antibody titers in 70% of vaccine recipients (44). Modern methods for producing sufficient amounts of good manufacturing practice (GMP)-grade recombinant NA have been developed (57). So far, one clinical trial evaluating trivalent inactivated vaccines (TIV) spiked with recombinant N2 followed by an H3N2 challenge has been performed. The results were reported in a conference abstract and describe a positive outcome of this study, but the results have not been published in a peer-reviewed journal (58). Additional clinical trials that evaluate NA-only vaccines or NA-spiked inactivated vaccines are urgently needed to find formulations that induce optimal anti-NA immunity. Importantly, no GMP-produced purified NA antigen is currently available, and it will be needed for clinical trials.

### Is correctly folded, functional NA required to induce protective immune responses?

Whether functional NA is required to induce protective immune responses is connected to the previous point. Sultana et al. have observed that there is a correlation between the enzymatic activity (as a proxy of correct folding) of NA in vaccines and its ability to induce NI antibody responses (53). Several anti-NA monoclonal antibodies (MAbs) that have been isolated and were found to be protective in animal models have been shown to bind to conformational epitopes as well (16). However, this is not completely clear, and it might be possible to induce protective anti-NA responses with denatured NA. In turn, we assume that the antibody response is best measured using structurally intact NA, but other reagents, even peptides, might be useful to measure protective antibody responses in some cases. This is a very important point, and further research is needed to clarify this question.

### Breadth of the anti-NA response.

A considerable breadth of both monoclonal and polyclonal antibody responses against NAs has been reported. Typically, cross-reactivity to some extent is observed within a subtype (e.g., within N1) (18, 22, 23, 31, 59) but not across subtypes (e.g., N1 to N2) (23). The exception is an epitope reported to induce reactivity across all NA subtypes, including cross-reactivity between influenza A and B NAs, albeit with low inhibiting and protective effects (60, 61). Thus, data about the breadth of the NA response is limited and has mostly been generated from animal models. Human data, ideally based on monoclonal antibodies, needs to be generated in order to conclusively understand NA’s potential as a broadly protective antigen.

### Antigenic drift.

Antigenic drift occurs in NA but has largely been ignored. Preliminary studies describe the drift rates as discordant and somewhat lower than the drift rates of HA (34, 62, 63). While some of these data have been generated using H6N*X* reagents (the *X* stands for the NA subtype) for NI assays, older data are based on wild-type virus reagents and are less reliable. To better understand the antigenic drift of N1, N2, and influenza B NAs, it is necessary to extend antigenic cartography (for which NA-specific antisera against matched and mismatched strains are needed), characterize epitopes using human sera and MAbs, and include NA sequencing into routine surveillance.

### Is N2 more immunogenic than N1?

Several studies have shown more robust immunity to N2 than to N1 in humans (23, 53, 64, 65). It is unclear whether this is an artifact of the assays used or whether this is caused by an inherent difference in terms of protein stability or immunogenicity.

### Mucosal immunity.

Our knowledge about serum antibody responses to NA is limited, and even less is known about mucosal antibody responses to NA. It is unclear to what level mucosal antibodies against N1, N2, and influenza B NAs are induced by infection, inactivated vaccines, and LAIV and how long-lived these responses are. Given the role of NA in trafficking virus to and from the site of infection in the respiratory tract, these are very relevant for protection in humans, and therefore NA-specific immunity at the mucosal surface is a critical gap in our knowledge.

### Mechanisms of action of anti-NA antibodies.

Many antibodies that target NA can inhibit its enzymatic function. This might inhibit viral egress from infected cells. The role of anti-NA immunity in inhibiting the release of incoming virions trapped by mucins on mucosal surfaces is less clear. Also, the role of anti-NA immunity in virus aggregation is unclear. The last two points are important, since NA-based immunity, if robust enough, might have a significant impact on the transmission of the virus, which might increase the effectiveness of the influenza virus vaccine even in areas with low vaccine coverage. Finally, it has been reported that some of the currently circulating H3N2 viruses use their NA instead of their HA for attachment to cells (66–68). In this case, anti-NA antibodies might also have HI activity.

### Contribution to receptor specificity.

NA binds to sialic acid in order to cleave it. This modulates the receptor binding specificity of viral particles, but our knowledge about this activity and about the receptor specificities of different NAs is limited. It is also unclear how this might be modulated by anti-NA immunity.

### Effector functions of anti-NA antibodies.

Antibody effector functions, like antibody-dependent cell-mediated cytotoxicity (ADCC) and antibody-dependent cellular phagocytosis (ADCP), have been found to be important mechanisms of action of broadly protective anti-HA antibodies (69–72). Very little is known about the effector function of anti-NA antibodies, and this needs to be explored. Interestingly, binding of HA to sialic acid on effector cells enhances the ADCC activity of anti-HA MAbs (73, 74). It can be hypothesized that the enzymatic activity of NA, which cleaves sialic acid, counteracts this mechanism. Inhibition of the NA enzymatic activity through antibodies might therefore mediate effector functions on its own and enhance effector functions of anti-HA antibodies by blocking the NA activity (75–77). Studies to address these questions are urgently needed.

### T-cell epitopes.

Early studies demonstrated that NA contains a number of CD4^+^ and CD8^+^ T-cell epitopes in mice (78, 79). NA is also the target of human CD4^+^ and CD8^+^ T cells, with epitopes listed in the Immune Epitope Database (IEDB; http://www.iedb.org/). We therefore expect a robust T-cell-dependent antibody response when NA is used as a stand-alone vaccine, with potential to induce cytotoxic CD8^+^ T cells when delivered with appropriate adjuvants, as live attenuated vaccine or by a viral vector. Studies to evaluate NA-specific T-cell responses are needed to design optimal NA-based vaccines.

### NA glycosylation.

NA is a glycoprotein with several N-linked glycosylation sites. Some of them are conserved, and others change over time. The role of glycosylation in enzymatic activity, immune evasion, and antigenic drift deserves further study.

### Modulation of immune mediators.

We know that NA can activate transforming growth factor β (TGF-β) during infections (80, 81). The underlying mechanism and the consequences for the host are not well understood. At low virus doses, NA enhances the ability of dendritic cells to initiate a T-cell response, but at high virus doses, NA contributes to the induction of Th2-type cytokines that may be associated with adverse events (82–85). Whether an NA-based vaccine will activate TGF-β and influence the quality of the immune response needs to be examined. In addition, studies counteracting these mechanisms during infection with NA-targeting antibodies should be conducted to determine the overall benefit of NA immunity to the host. The functional consequences of NA activity on initiation of the immune response and of NA-specific antibodies on effector functions during virus challenge certainly need to be evaluated.

## WHICH ASSAYS EXIST, AND WHAT ARE THEIR LIMITATIONS?

Several assays to analyze the NA contents of vaccines, the NA integrity of vaccine preparations, and anti-NA antibody responses have been designed and are currently in use. For immune assays, it should be noted (as discussed above) that we lack a good understanding of which readouts correlate with protection.

### Assays to determine the NA contents of vaccines.

The simplest, although only semiquantitative, way of measuring the NA contents of influenza virus vaccines is by reducing SDS-PAGE followed by Western blotting using a protein standard and strain/subtype-specific polyclonal or monoclonal antibody (23). While this method does not allow for the assessment of the structural integrity of NA (besides its very obvious proteolytic degradation), it can be an easy way to get a good estimate of how much NA is present in the vaccine, and it can be used to compare different vaccine preparations, formulations, and lots. A better method is the use of quantitative enzyme-linked immunosorbent assays (ELISAs) to directly measure NA concentrations. Depending on the capture and/or detection antibody chosen, these assays can be used to actually measure correctly folded NA (86). A chip-based VaxArray (87) assay using the same principle is under development (personal communication, Kathy Rowlen, InDevR). In addition, high-performance liquid chromatography (HPLC)- and mass spectrometry-based methods have been developed, but they are more burdensome and do not necessarily indicate protein integrity (52, 88). Finally, NA activity assays might be of value as well in analyzing the quality of NAs present in vaccines, since only tetrameric NA has strong sialidase activity (53). However, these assays are useful only with monovalent vaccines, because they cannot specifically measure the activity of each strain/subtype in a multivalent vaccine.

### Assays to characterize the anti-NA immune response.

ELISAs are a relatively easy way to measure immune responses to NA. They can be performed with serum or other bodily fluids (e.g., nasal washes); with different secondary antibodies, the immune response can be dissected into different IgG subtypes and IgA, sIgA, and IgM responses. The assay can be performed in a quantitative way by measuring endpoint titers or by performing an area under the curve analysis. To evaluate NA responses, the use of recombinant, tetrameric, glycosylated, and enzymatically active NA as the substrate is likely the best choice. While ELISAs yield binding data, they do not inform about the functionality of the measured antibody response. However, the ELISA titers usually correlate well with NI titers measured in functional assays (64). Several assays to measure the enzymatic activity of NA exist. Many of these assays are based on cleavage of a small molecule that subsequently leads to the development of a signal (89) (http://apps.who.int/iris/bitstream/10665/44518/1/9789241548090_eng.pdf). However, unlike terminal sialic acid, which is attached to glycans on bulky proteins, these small molecules have easy access to the active site of the NA. To show NI activity in a small-molecule-based assay, an antibody needs to bind very close to the active site and/or inhibit the active site allosterically. However, anti-NA MAbs can also block NA activity by steric hindrance of the natural substrate-NA interaction, since the substrate, terminal sialic acid, is usually attached to a large bulky glycoprotein or glycolipid. Therefore, the enzyme-linked lectin assay (ELLA) that uses fetuin, a highly sialylated glycoprotein, as the substrate was developed to measure NI antibody titers in a more realistic way (20, 90). This assay is relatively simple and can be performed in almost any laboratory that has access to a plate reader. The key reagent for the ELLA is the target NA to which NI activities of sera or MAbs should be measured. The NA can be used in the form of a virus or as purified NA. If wild-type virus is used, the fact that antibodies that bind to the HA head domain exhibit strong NI activity due to steric hindrance needs to be taken into account as well (19, 91). While it is sometimes useful to know the potential of serum to inhibit NA activity regardless of whether it is mediated by binding to NA or other targets (92), it is usually of interest to measure NA-specific inhibition. To accomplish this, H6N*X* and/or H7N*X* reassortant viruses are commonly used to reduce the impact of anti-HA antibodies on the assays (20, 63, 91). However, it needs to be noted that antistalk antibodies that broadly bind to HAs (including H6) might interfere with the assay in some cases too (64). Virus-like particles (VLPs) with mismatched HAs might be used for NI assays as well (93). Another possibility is to perform the NI assay with recombinant NA, which eliminates issues with non-NA-specific inhibition (94, 95). However, soluble NA might have enzyme kinetics different from that of membrane-bound NAs, and it seems that HA on virions also plays an important part in bringing NA and the substrate in the assay into close proximity (96). Pseudotyped viruses and detergent-disrupted viruses have also been used as antigens to measure NA inhibition antibody titers (50, 97). The ELLA with H6N*X* viruses (or viruses with other exotic HAs, e.g., H7N*X*) is, however, recommended as the gold standard assay for measuring NI activity. Finally, NA-based assays to measure effector functions of antibodies might be useful in the future (16, 75). These assays could be designed by following protocols of current assays for measuring effector functions (e.g., the ADCC and ADCP reporter assays that are in wide use) with target cells that have been transfected to express NA only (instead of infected cells).

## WHAT KIND OF REAGENTS HAVE BEEN CREATED, AND FROM WHERE CAN THEY BE SOURCED?

Reagents for the assays listed above are still relatively scarce compared to reagents for HA-based assays. Here, we list the available reagents as well as their advantages and disadvantages. Main characteristics and sources can be found in Table 1. As mentioned above, it has become good practice to use H6N*X* viruses (or other viruses that express mismatched HA subtypes) for NI assays to reduce the influence of non-NA antibodies on the assay. These viruses are typically generated by reverse genetics using an avian H6 HA in combination with the target NA. Relatively large panels of H6N1 and H6N2 viruses for ELLAs have been developed and can be requested from members of this group (reagents that target other subtypes of NA, like H6N9, exist as well). While analysis of influenza B viruses was restricted to wild-type viruses until recently, H6NB viruses have now also been rescued (64). While an H6 HA is commonly used for these reassortants, other exotic HA subtypes can be used as well (e.g., H7N*X*) but may result in the need to treat the serum samples to remove HA-specific inhibitors (63). However, two caveats with these viruses need to be kept in mind. First, stalk-reactive antibodies might interfere with the assay and induce a low level of NI background (19, 64). Second, when wild-type viruses and H6N*X* viruses with the same NAs were tested *in vitro* with MAbs, the NI activities differed slightly between the viruses (64). This might be caused by different HA/NA ratios between wild-type and H6N*X* viruses or by differences in the affinities of the HAs toward the substrate (see above), and it is unclear whether this also occurs with polyclonal sera. Nevertheless, H6N*X* reassortant viruses are currently seen as the best reagent to measure NI activity (except when testing sera from animals that have been vaccinated with NA-only vaccines). Recombinant NA can be a very useful reagent for ELISAs and other assays, as well as for vaccination studies, to create NA-specific antisera. Recombinant NA should ideally be expressed as a tetramer with fully functional enzymatic activity to preserve as much of the antigenic structure as possible (23, 94, 98, 99). Typically, only the head domain of the NA is expressed as a fusion protein with a tetramerization domain in insect cells or mammalian cells (100). A production process for full-length NA from insect cells has also been developed but involves considerable downstream processing (57). NA expressed in bacterial systems is unlikely to be correctly folded and representative of virus-derived NA. Of note, even when expressed under the best conditions, some NAs are unstable in the form of their recombinant proteins. A selection of recombinant NA proteins is available commercially or from the International Reagent Resource (IRR) or BEI Resources. Larger panels might be requested from this group, and considerable efforts are made to make more NAs available, but expression and purification of NA are costly and need support. In addition, a larger number of MAbs and NA-specific polyclonal sera are needed as reagents for use in antigenic cartography, potency ELISAs, Western blot assays, competition assays, and positive controls in NI and serum ELISAs.

**TABLE 1:** Useful reagents for evaluation of NA contents of vaccines and NA-directed immunity

Reagent(s)	Assay(s)^a^	Comment	Availability^b^	Reference
H6N*X* viruses	NI	Viruses containing avian components (e.g., H6) might need specific permits for shipping and use	Available upon request from Hongquan Wan (FDA), Richard Webby (St. Jude Children’s Research Hospital), and Florian Krammer (Icahn School of Medicine at Mount Sinai)	20, 64, 90, 91
Fetuin	NI		Widely commercially available	
Horseradish peroxidase- labeled peanut agglutinin	NI		Widely commercially available	
Recombinant NA	ELISA, NI, and WB (as the standard) for NA-specific serum generation and NA binding studies	Only functional, glycosylated, tetrameric NA expressed in mammalian cells or insect cells should be used	Available from BEI Resources (https://www.beiresources.org/), International Reagent Resource (https://www.internationalreagentresource.org/), and commercial sources, and limited amounts are available upon request from Florian Krammer (Icahn School of Medicine at Mount Sinai); these reagents are difficult and costly to produce and are therefore available only in small quantities	23, 94, 98–100
Anti-NA MAbs	Capture ELISA for NA quantification, competition assays, and assay positive controls	Useful for different purposes, depending on the nature of the epitope recognized (linear versus conformation dependent)	Some MAbs are available upon request from Hongquan Wan (FDA), Florian Krammer (Icahn School of Medicine at Mount Sinai), and others; in general, there is a paucity of anti-NA MAbs, and additional ones against different strains and subtypes are needed	16, 18, 23, 35, 86
Anti-NA polyclonal sera	Positive controls, WB, and antigenic cartography	NA specific versus virus specific	Not widely available; there is a need to generate these reagents; very limited amounts for some strains are available from Ron Fouchier (Erasmus Medical Center)	63

a WB, Western blotting.

b These reagents might be requested, but availability is not guaranteed and might depend on legal restrictions, available resources, and funding to produce batches and other factors.

## CONCLUSIONS

NA is a fascinating protein that plays multiple essential roles in the influenza virus life cycle. In addition, there is evidence that NA is an important and protective antigen. In order to confirm that role and to harness NA-based immunity optimally to enhance the breadth of influenza virus vaccines and increase vaccine efficacy, studies that answer the questions stated above are urgently needed. In addition, clinical trials and observational studies that evaluate NA-based and/or NA-enhanced vaccines in humans are needed. These are major efforts, but we feel that they are necessary to reveal the full potential of influenza virus vaccines, both for currently licensed seasonal vaccines and for future broadly protective or universal influenza virus vaccines (101). To facilitate these efforts, the NAction! focus group was formed within the CEIRS network by experts in NA-based immunity and includes major stakeholders outside the CEIRS network. We invite other researchers with a strong interest in NA-based immunity to contribute to these efforts and/or join the group by contacting the CEIRS program officer, Marciela DeGrace (marciela.degrace@nih.gov), at NIAID.
